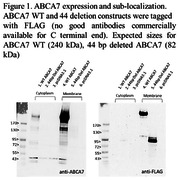# Uncovering the Role of an African‐specific *ABCA7* Frameshift Deletion on Lipid Metabolism and Alzheimer’s Disease

**DOI:** 10.1002/alz.092425

**Published:** 2025-01-03

**Authors:** Younji Nam, Brooke A. DeRosa, Charles G. Golightly, Shaina A. Simon, Jamie Arvizu, Aura M Ramirez, Patrice L. Whitehead, Larry D. Adams, Takiyah D. Starks, Holly N. Cukier, Michael L. Cuccaro, Jonathan L. Haines, Goldie S. Byrd, Gary Beecham, Derek M. Dykxhoorn, Juan I Young, Jeffery M. Vance, Margaret A. Pericak‐Vance

**Affiliations:** ^1^ John P. Hussman Institute for Human Genomics, University of Miami Miller School of Medicine, Miami, FL USA; ^2^ Dr. John T. MacDonald Foundation Department of Human Genetics, University of Miami Miller School of Medicine, Miami, FL USA; ^3^ John P. Hussman Institute for Human Genomics, University of Miami Miller School of Medicine, Miami, FL, USA, Miami, FL USA; ^4^ John P. Hussman Institute for Human Genomics, Miller School of Medicine, Miami, FL USA; ^5^ Dr. John T. Macdonald Foundation Department of Human Genetics, University of Miami Miller School of Medicine, Miami, FL USA; ^6^ Maya Angelou Center for Health Equity, Wake Forest University School of Medicine, Winston‐Salem, NC USA; ^7^ Department of Population and Quantitative Health Sciences, Institute for Computational Biology, Case Western Reserve University, Cleveland, OH USA; ^8^ John P. Hussman Institute for Human Genomics, Miami, FL USA

## Abstract

**Background:**

We previously identified a 44‐base pair deletion in (ATP‐binding cassette sub‐family A member 7) (*ABCA7*) that is significantly associated with Alzheimer’s disease (AD) in African Americans (AA), producing a frameshift mutation resulting in a truncated protein (p.Arg578Alafs). *ABCA7* is a lipid transporter across cellular membranes. While we have shown the mutant mRNA is present in neurons, whether it is translated into a stable protein is not known due to the lack of antibodies capable of recognizing the N‐terminus of endogenous *ABCA7*. The abrupt *ABCA7* translation due to the deletion may alter the lipid metabolism, which can be investigated using isogenic iPSC and differentiated models.

**Methods:**

We analyzed single‐cell RNAseq (scRNAseq) data from spheroid cultures of 12 individuals. We generated a recombinant version of the *ABCA7* gene with and without the deletion and bearing an N‐terminal flag tag which were transfected into HEK cells. We utilized CRISPR‐based genome editing using induced pluripotent stem cell (iPSC) lines from three independent AA‐cognitively unimpaired individuals (WT) to introduce the AA‐specific *ABCA7* homozygous or heterozygous deletions. These were differentiated using standard protocols in the HIHG iPSC core into monocultures of induced neuron‐like cells. C‐terminal antibodies were used to assess the presence of native *ABCA7* in HEK cells.

**Results:**

Examination of HEK cells revealed no detectable native *ABCA7*. The truncated *ABCA7*‐tagged protein appeared stable and localized in the plasma membrane as seen for the wild‐type protein (Figure 1). ScRNAseq confirmed that expression of *ABCA7* is highest in neurons, identifying them as the iPSC differentiated cell of choice. We successfully generated both homozygous and heterozygous deletion isogenic lines in the three WT iPSC lines. Differentiated neurons from these isogenic lines have a normal phenotype, allowing for functional assays and lipidomic studies.

**Conclusion:**

The truncated protein p.Arg578Alafs appears to be expressed and stable in HEK cells, and surprisingly located in the plasma membrane despite the absence of most transmembrane domains. Our three isogenic iPSC pairs will be a great resource for studying the pathogenic effects of the *ABCA7* truncation in differentiated cells.